# Experimental Transmission of Karshi (Mammalian Tick-Borne Flavivirus Group) Virus by *Ornithodoros* Ticks >2,900 Days after Initial Virus Exposure Supports the Role of Soft Ticks as a Long-Term Maintenance Mechanism for Certain Flaviviruses

**DOI:** 10.1371/journal.pntd.0004012

**Published:** 2015-08-18

**Authors:** Michael J. Turell

**Affiliations:** Virology Division, U.S. Army Medical Research Institute of Infectious Diseases, Fort Detrick, Maryland, United States of America; North Carolina State University, UNITED STATES

## Abstract

**Background:**

Members of the mammalian tick-borne flavivirus group, including tick-borne encephalitis virus, are responsible for at least 10,000 clinical cases of tick-borne encephalitis each year. To attempt to explain the long-term maintenance of members of this group, we followed *Ornithodoros parkeri*, *O*. *sonrai*, and *O*. *tartakovskyi* for >2,900 days after they had been exposed to Karshi virus, a member of the mammalian tick-borne flavivirus group.

**Methodology/Principal Findings:**

Ticks were exposed to Karshi virus either by allowing them to feed on viremic suckling mice or by intracoelomic inoculation. The ticks were then allowed to feed individually on suckling mice after various periods of extrinsic incubation to determine their ability to transmit virus by bite and to determine how long the ticks would remain infectious. The ticks remained efficient vectors of Karshi virus, even when tested >2,900 d after their initial exposure to virus, including those ticks exposed to Karshi virus either orally or by inoculation.

**Conclusions/Significance:**

*Ornithodoros* spp. ticks were able to transmit Karshi virus for >2,900 days (nearly 8 years) after a single exposure to a viremic mouse. Therefore, these ticks may serve as a long-term maintenance mechanism for Karshi virus and potentially other members of the mammalian tick-borne flavivirus group.

## Author Summary

Members of the mammalian tick-borne flavivirus group, including tick-borne encephalitis virus, remain a significant cause of human disease and are responsible for at least 10,000 clinical cases of tick-borne encephalitis each year. One of the principal questions in their epidemiology is how they persist from year to year in a given area. To attempt to explain the long-term maintenance of members of this group, we exposed *Ornithodoros parkeri*, *O*. *sonrai*, and *O*. *tartakovskyi* ticks to Karshi virus, a member of the mammalian tick-borne flavivirus group. Ticks were exposed to Karshi virus either by allowing them to feed on viremic suckling mice or by intracoelomic inoculation. To determine their ability to maintain the virus for an extended period of time and to transmit Karshi virus, ticks were allowed to feed individually on suckling mice after various periods of extrinsic incubation. Ticks exposed to Karshi virus, either orally or by inoculation, remained efficient vectors of Karshi virus, even when tested >2,900 days (approximately 8 years) after their initial exposure to virus. Therefore, these ticks may serve as a long-term maintenance mechanism for Karshi virus and potentially other members of the mammalian tick-borne flavivirus group.

## Introduction

Karshi virus is a member of the mammalian tick-borne flavivirus group (genus *Flavivirus*, family *Flaviviridae*) [1]. Members of this group include tick-borne encephalitis virus (including subtypes Central European encephalitis virus (CEEV) and Russian spring-summer encephalitis virus (RSSEV), Omsk hemorrhagic fever virus, Langat virus (LGTV), Alkhurma hemorrhagic fever virus, Kyasanur Forest disease virus (KFDV), Powassan virus (POWV), Royal Farm virus, Karshi virus, Gadgets Gully virus, and Louping ill virus [1,2]. This group of viruses, also known as the TBEV serocomplex [1,3], are responsible for at least 10,000 clinical cases of tick-borne encephalitis each year [4]. A second group of tick-borne flaviviruses is known as the seabird tick-borne flavivirus group [5]. Although a member of the mammalian tick-borne flavivirus group, Karshi virus is not known to cause disease in humans [5]. However, its close relationship to both POWV and KFDV indicated that it should be capable of causing disease in humans [1,2].

The natural transmission cycle of the mammalian tick-borne flavivirus group involves ixodid ticks and rodents, with *Ixodes ricinus* and *I*. *persulcatus* being the principal vectors of CEEV and RSSEV viruses, respectively [6,7]. This cycle is essentially identical to that for the Lyme disease spirochete, *Borrelia burgdorferi*, in *I*. *scapularis*. In the Lyme disease cycle, the mouse, *Peromyscus leucopus*, remains infectious for several months [8]. Therefore, once a mouse becomes infected by being fed upon by an infectious nymphal tick, it would continue to expose larval and nymphal ticks to the spirochete for months. However, because viremias in rodents exposed to members of the mammalian tick-borne flavivirus group are transient, often lasting only a few days [9,10], the timing of nymphal and larval attachment becomes critical. If infectious nymphal ticks attach too early in the season, the viremia in the rodent will have ended prior to the attachment of the larval ticks. Unlike these Ixodid (hard) ticks that normally attach for 2–13 days to complete a blood meal and only feed once during the larval, nymphal, and adult stages [11], members of the genus *Ornithodoros* attach and complete feeding usually within 10–30 min and most complete feeding within an hour [12]. Also, these ticks will feed multiple times both as nymphs and as adults, often live in rodent borrows, and can live about 20 years [12,13]. Previous studies indicate that *Ornithodoros* spp. ticks are able to become infected and transmit members of the mammalian tick-borne flavivirus group [14–16] as well as other pathogens [12]. Because of their long life span and repeated feedings, they can remain infectious for an extended period of time. *Ornithodoros tholozani* were shown to transmit *Borrelia persica* (a causative agent of relapsing fever) for at least 13 years after a single exposure [13] and field-collected *O*. *turicata* were able to transmit *B*. *recurrentis* (repsorted as *Spirochaeta recurrentis*) for at least 6.5 years [17]. In addition, many *Ornithodoros* spp. ticks are considered to be nidicolous, i.e., living in close association with their vertebrate hosts such as living in rodent burrows [18]. *Onithodoros sonrai* are found in burrows of many rodent genera in Senegal and western Africa [19]; *O*. *tartakovskyi*, which is widely distributed in central Asia from Iran to the Xinjiang Province in western China are found in burrows of various rodent species, but primarily the great gerbil, *Rhombomys opimus*, [20,21]; and *O*. *parkeri* found in the western portions of the United States and Canada, is associated with numerous rodent species, but primarily prairie dogs [22,23]. To determine the potential for these ticks to serve as a long-term maintenance mechanism for these viruses, we evaluated the potential for *O*. *sonrai*, *O*. *parkeri*, and *O*. *tartakovskyi* ticks to transmit Karshi virus over an extended period of time.

## Methods

### Ticks

We used three species of *Ornithodoros* ticks. These included a laboratory colony of *O*. *sonrai* derived from wild-caught specimens excavated from mammal burrows in the Bandia Forest of Senegal in 1989 [15]. No virus was detected upon examination of parental ticks from this colony. Georgia Southern University provided a colony of *O*. *parkeri* derived from specimens captured in Spicer City, CA, in 1965. The National Institute of Allergy and Infectious Diseases provided a laboratory colony of *O*. *tartakovskyi*. All three colonies were maintained as described by Durden et al. [24].

### Virus and Virus Assays

We used the U2-2247 strain of Karshi virus. It had been passaged once in Vero cells and once in suckling mice before use in these experiments. Serial dilutions of blood, brain, and tick samples were tested for virus by plaque assay on confluent monolayers of 2- to 3-d-old primary chicken embryo cells or by subcutaneous inoculation into 2- to 4-d-old suckling mice. The identity of the original virus, and virus recovered from ticks and mice, was confirmed by a Karshi-specific quantitative real-time Real Time- polymerase chain reaction (PCR) assay and by direct sequencing of the PCR products [16,25].

### Experimental Design

One-day-old suckling mice (BALB/c strain) were inoculated intraperitoneally with 10^6.3^ suckling mouse lethal dose_50_ (SMLD_50_) units of Karshi virus. Two or 3 days after inoculation, a Karshi virus-inoculated mouse was placed in a cage containing ~50 *O*. *sonrai*, *O*. *parkeri*, or *O*. *tartakovskyi* ticks at various stages of development (larvae through adult, but predominately early nymphs). After the ticks had been allowed to attach to the mouse for about 5 min, the mouse was removed and a second virus-inoculated mouse was added to the cage. This was repeated for up to three mice for each species of tick used in this study. The ticks were allowed to feed on the virus-inoculated mouse for about 2 h. At that time, those ticks that had attached and did not feed were removed and discarded. Each mouse was then euthanized with CO_2_ and blood was collected by cardiac puncture. Blood was mixed 1:10 in diluent (Medium 199 with Earle’s salts containing 10% heat-inactivated fetal bovine serum and 5 μg of amphotericin B, 50 μg of gentamicin, 100 units of penicillin, and 100 μg of streptomycin per ml and 0.075% NaHCO_3_) and frozen at -70°C until tested to determine the viremia at the time of tick feeding. The engorged ticks were placed in a cage maintained at room temperature (~20°C) until tested for either infection or for the ability to transmit virus by bite. For each species, some of the ticks that had not attached to a virus-inoculated mouse were inoculated intracoelomically with 10^4^ SMLD_50_ (10^7.5^ SMLD_50_/ml) of the same virus strain that had been used to infect the mice [26]. These inoculated ticks were treated in the same manner as the engorged ticks, except that the inoculated *O*. *parkeri* were maintained in an incubator maintained at 26°C rather than at ambient air temperature.

To determine transmission rates, virus-exposed ticks were allowed to feed for up to 2 hours on naive suckling mice (either BALBc or Swiss Webster) individually, i.e., one tick per mouse. These suckling mice were marked by subcutaneous inoculation of India ink, returned to their dam, and then monitored daily over the next 21 d for signs of viral infection. Each litter contained one or two suckling mice that were either unexposed to ticks or were fed upon by a tick from the uninfected colony to serve as negative controls. Moribund mice were euthanized with CO_2_, and brain samples were obtained from a subset of them and then triturated (1:10) in diluent and frozen at -70°C until tested for virus. In most of the tick transmission trials, ticks were caged individually in plastic vials (12 ml, about half filled with washed sea sand) after feeding on the mice. Many of these same ticks were tested multiple times over the following 8 years for their ability to transmit virus by bite.

### Ethics Statement

Research was conducted under an IACUC approved protocol in compliance with the Animal Welfare Act, PHS Policy, and other Federal statutes and regulations relating to animals and experiments involving animals. The facility where this research was conducted is accredited by the Association for Assessment and Accreditation of Laboratory Animal Care, International and adheres to principles stated in the Guide for the Care and Use of Laboratory Animals, National Research Council, 2011. The USAMRIID IACUC approved these studies.

## Results

### Oral Exposure Experiments

Viremias in the suckling mice at the time of the tick feedings ranged from 10^6.5^ to 10^6.7^ SMLD_50_/ml. When allowed to feed on a susceptible mouse ≤94 days after the initial blood meal, transmission was very inefficient, with none of 17 ticks transmitting virus to the mice (Table 1). However, when tested ≥105 days after the initial feeding, at least 60% of the ticks that had fed on a mouse with a viremia about 10^6.5^ SMLD_50_/ml transmitted virus, regardless of tick species, including several ticks that failed to transmit virus when allowed to feed at days 59–94 after virus exposure. When ticks that had transmitted virus on one occasion were allowed to feed on a second mouse at some point in the future, nearly all of them (86%, n = 14) transmitted each time they were allowed to feed. Each of the species transmitted virus the last time it was tested, and all species transmitted virus for at least 2,000 days (Table 1). Data for each transmission attempt is provided in S1 Table.

**Table 1:** Transmission of Karshi virus by *Ornithodoros* ticks after feeding on mice with a viremia about 10^6.5^ SMLD_50_/ml of blood.

		Transmission rates (≥105 days)		
Species	Trans. Rate (≤94 days)^*a*^	By tick^*b*^	All feeds^*c*^	Day last transmission^*d*^
*O*. *parkeri*	n.t.	85 (13)	97 (29)	2905
*O*. *sonrai*	0 (15)	56 (32)	84 (31)	2905
*O*. *tartakovsky*	0 (6)	67 (6)	100 (6)	2109

n.t., not tested

^*a*^Percentage transmitting virus by bite (number of ticks feeding). Each feeding consisted of one tick on one mouse.

^*b*^Data presented for each tick, i.e., percentage of the ticks that transmitted virus (number of individual ticks fed)

^*c*^Data presented for all feedings for ticks that had transmitted virus, i.e., percentage of the ticks that transmitted virus (number of feeding attempts, including multiple attempts by the same tick on different days).

^*d*^Last day on which tick successfully transmitted virus.

### Inoculation Experiments

For both *O*. *sonrai* and *O*. *tartakovskyi*, five of six ticks transmitted virus by bite when tested 43 days after inoculation with Karshi virus (Table 2). However, all 34 ticks (eight *O*. *parkeri*, 11 *O*. *sonrai*, and 15 *O*. *tartakovskyi*) tested at ≥64 days after inoculation transmitted Karshi virus by bite. Data for each transmission attempt is provided in S2 Table.

**Table 2:** Transmission of virus by *Ornithodoros* ticks after intracoelomic inoculation of 10^4^ SMLD_50_ of Karshi virus.

			Transmission rates (≥63 days)		
Species	Holding temperature	Trans. Rate (day 43)^a^	By tick^b^	All feeds^c^	Day last transmission^d^
*O*. *parkeri*	26°C	n.t.	100 (6)	100 (8)	1910
*O*. *sonrai*	20°C	83 (6)	100 (11)	100 (13)	252
*O*. *tartakovsky*	20°C	83 (6)	100 (15)	100 (21)	2106

n.t., not tested

^*a*^Percentage transmitting virus by bite (number of ticks feeding). Each feeding consisted of one tick on one mouse.

^*b*^Data presented for each tick, i.e., percentage of the ticks that transmitted virus (number of individual ticks fed).

^*c*^Data presented for all feedings, i.e., percentage of the ticks that transmitted virus (number of feeding attempts, including multiple attempts by the same tick on different days).

^*d*^Last day on which transmission was attempted.

These 34 ticks took a total of 43 blood meals from susceptible mice and transmitted virus in each case (Table 2). Individuals in each species transmitted virus the last time that species was tested, with the final transmission occurring >2,100 days after the tick had initially been inoculated with Karshi virus.

## Discussion

*Ornithodoros* spp. ticks were able to transmit Karshi virus for >2,900 days (nearly 8 years) after a single exposure to a viremic mouse. Therefore, these ticks may serve as a long-term maintenance mechanism for Karshi virus and potentially other members of the mammalian tick-borne flavivirus group. This study was a continuation of a study [16] that examined the potential for these *Ornithodoros* ticks to transmit Karshi virus, but that original study only followed the tick for 3 years.

Traditionally, viruses in the mammalian tick-borne flavivirus group have been associated with ixodid ticks, *I*. *ricinis* and *I*. *persulcatus* in Europe and Asia, respectively [6,7] and with *I*. *cookei* and *I*. *scapularis* in the Americas [27]. Larval and nymphal ticks are exposed to virus when overwintering infected nymphal ticks feed on naïve rodents in the spring. These ticks can also be infected by co-feeding with an infected tick [28], regardless of the immune status of the rodent [29]. However, transmission by co-feeding on an immune rodent was only about 10% as efficient as co-feeding on an immunologically naïve rodent when the two ticks were not immediately collocated [29]. Given the relatively short period of viremia for these viruses in their rodent hosts [9,10], one could hypothesize that this cycle would be too inefficient to maintain these viruses for many years in the same location. However, if a rodent became infected after being fed upon by an infectious tick and then went back to its burrow, it could potentially expose many of the *Ornithodoros* ticks living in that burrow. When that rodent died, or was killed by a predator, the burrow would remain vacant until discovered by a new rodent. Individual *Ornithodoros* ticks can remain viable for up to 4 years between feedings [13,30,31] and can survive for 10–20 years [13,32–34]. In addition, this study observed transmission of Karshi virus for up to 8 years post infection. Thus, ticks present in the vacant rodent burrow could remain a source of virus for many years. When a new rodent entered that burrow and was fed upon by the infected *Ornithodoros* ticks, the rodent would become infected and all the ixodid ticks present on that rodent exposed to virus. These ixodid ticks could then spread the virus to other rodents and to larger mammals including humans.

*Ornithodoros* ticks have a wide distribution, with species found in much of the range of the mammalian tick-borne flaviviruses [35,36]. However, there are regions where members of this virus complex are found, but for which members of the genus *Ornithodoros* have not been described, i.e., the northeastern US for Powasson virus and deer tick virus, and parts of the northern range of the mammalian tick-borne flaviviruses in Eurasia. Therefore, other methods must exist for the perpetuation of these viruses in those areas.

Experimental studies on members of the mammalian tick-borne flavivirus group have focused on ixodid ticks. However, several members of this and the closely related seabird tick-borne flaviviruses group have been isolated from naturally occurring *Ornithodoros* ticks. These include Karshi virus [37], KFDV [38], Alkhurma hemorrhagic fever virus [39], Meaban virus [40], and Saumarez Reef virus [41].

Therefore, the susceptibility of *O*. *parkeri*, *O*. *sonrai*, and *O*. *tartakovskyi* to infection with Karshi virus; their ability to transmit this virus for extended periods (at least 2,905 days); their long life span; and the isolation of several members of both the mammalian and seabird tick-borne flavivirus groups from *Ornithodoros* ticks indicate that *Ornithodoros* species should be studied as potential long-term reservoir hosts for members of the tick-borne flavivirus groups.

## Supporting Information

S1 Table The results are presented for each transmission attempt for individual *O*. *parkeri*, *O*. *sonrai*, and *O*. *tartakovsky* tested at various days after being orally exposed to Karshi virus. (DOCX)

S2 Table The results are presented for each transmission attempt for individual *O*. *parkeri*, *O*. *sonrai*, and *O*. *tartakovsky* tested at various days after being inoculated with Karshi virus. (DOCX)
