# Supplementary material for: Experimental Transmission of Karshi (Mammalian Tick-Borne Flavivirus Group) Virus by Ornithodoros Ticks >2,900 Days after Initial Virus Exposure Supports the Role of Soft Ticks as a Long-Term Maintenance Mechanism for Certain Flaviviruses
Source: PLoS Negl Trop Dis. 2015 Aug 18;9(8):e0004012. doi: 10.1371/journal.pntd.0004012 (PMC4540281; doi:10.1371/journal.pntd.0004012)
Supplement: S1 Table — (DOCX) [file pntd.0004012.s001.docx]

| Supplemental Table 1a. Transmission attempts for individual *O. parkeri* tested at various days after being orally exposed to Karshi virus | | | | | | | | | | | | | | | | | |
| --- | --- | --- | --- | --- | --- | --- | --- | --- | --- | --- | --- | --- | --- | --- | --- | --- | --- |
| Tick  number | Date  Inoc. | Day | Trans.  Y/n | |  | | Day | Trans.  Y/n |  | Day | Trans.  Y/n |  | Day | Trans.  Y/n |  | Day | Trans.  Y/n |
| 517-101-1 | 19 June 2003 | 210 | Y | |  | | 601 | Y |  | 493 | Y |  | 1958 | Y |  | 2194 | Y |
| 517-101-2 | 19 June 2003 | 210 | Y | |  | | 601 | Y |  | 1958 | Y |  | 2905 | Y |  |  |  |
| 517-101-3 | 19 June 2003 | 210 | Y | |  | |  |  |  |  |  |  |  |  |  |  |  |
| 517-101-4 | 19 June 2003 | 210 | Y | |  | |  |  |  |  |  |  |  |  |  |  |  |
| 517-102-1 | 19 June 2003 | 210 | Y | |  | | 1294 | Y |  | 2905 | Y |  |  |  |  |  |  |
| 517-102-2 | 19 June 2003 | 210 | Y | |  | | 540 | Y |  | 1959 | Y |  | 2194 | Y |  |  |  |
| 517-102-3 | 19 June 2003 | 210 | Y | |  | | 540 | Y |  | 2905 | Y |  |  |  |  |  |  |
| 517-102-4 | 19 June 2003 | 210 | Y | |  | |  |  |  |  |  |  |  |  |  |  |  |
| 517-103-1 | 19 June 2003 | 210 | n | |  | |  |  |  |  |  |  |  |  |  |  |  |
| 517-103-2 | 19 June 2003 | 210 | n | |  | |  |  |  |  |  |  |  |  |  |  |  |
| 517-103-3 | 19 June 2003 | 210 | Y | |  | | 2194 | n |  |  |  |  |  |  |  |  |  |
| 517-103-4 | 19 June 2003 | 210 | Y | |  | | 1958 | Y |  |  |  |  |  |  |  |  |  |
| 517-103-5 | 19 June 2003 | 210 | Y | |  | | 493 | Y |  | 2194 | Y |  |  |  |  |  |  |
|  |  |  |  | |  | |  |  |  |  |  |  |  |  |  |  |  |
| Supplemental Table 1b. Transmission attempts for individual *O. sonrai* tested at various days after being orally exposed to Karshi virus | | | | | | | | | | | | | | | | | |
| 517-601-1 | 12 Dec 2003 | 41 | n | |  | | 126 | n |  |  |  |  |  |  |  |  |  |
| 517-601-2 | 12 Dec 2003 | 41 | n | |  | | 126 | n |  |  |  |  |  |  |  |  |  |
| 517-601-3 | 12 Dec 2003 | 41 | n | |  | |  |  |  |  |  |  |  |  |  |  |  |
| 517-601-4 | 12 Dec 2003 | 41 | n | |  | | 126 | n |  |  |  |  |  |  |  |  |  |
| 517-601-5 | 12 Dec 2003 | 41 | n | |  | |  |  |  |  |  |  |  |  |  |  |  |
| 510-601-1 | 22 Jan 2003 | 64 | n | |  | | 203 | Y |  |  |  |  |  |  |  |  |  |
| 510-601-2 | 22 Jan 2003 | 64 | n | |  | | 203 | n |  |  |  |  |  |  |  |  |  |
| 510-601-3 | 22 Jan 2003 | 64 | n | |  | |  |  |  |  |  |  |  |  |  |  |  |
| 510-601-4 | 22 Jan 2003 | 64 | n | |  | | 203 | Y |  |  |  |  |  |  |  |  |  |
| 510-601-5 | 22 Jan 2003 | 64 | n | |  | | 203 | n |  | 314 | n |  | 1102 | n |  | 2108 | n |
| 510-601-6 | 22 Jan 2003 | 64 | n | |  | | 203 | Y |  |  |  |  |  |  |  |  |  |
| 510-401-1 | 21 Jan 2003 | 65 | n | |  | | 314 | n |  |  |  |  |  |  |  |  |  |
| 510-401-2 | 21 Jan 2003 | 65 | n | |  | |  |  |  |  |  |  |  |  |  |  |  |
| 510-401-3 | 21 Jan 2003 | 65 | n | |  | |  |  |  |  |  |  |  |  |  |  |  |
| 510-401-4 | 21 Jan 2003 | 65 | n | |  | |  |  |  |  |  |  |  |  |  |  |  |
| 517-303-1 | 19 June 2003 | 105 | n | |  | |  |  |  |  |  |  |  |  |  |  |  |
| 517-303-2 | 19 June 2003 | 105 | Y | |  | |  |  |  |  |  |  |  |  |  |  |  |
| 517-603-1 | 12 Dec 2003 | 126 | n | |  | |  |  |  |  |  |  |  |  |  |  |  |
| 517-603-2 | 12 Dec 2003 | 126 | n | |  | |  |  |  |  |  |  |  |  |  |  |  |
| 517-604-1 | 12 Dec 2003 | 126 | Y | |  | | 365 | Y |  |  |  |  |  |  |  |  |  |
| 517-604-2 | 12 Dec 2003 | 126 | Y | |  | | 365 | Y |  |  |  |  |  |  |  |  |  |
| 517-604-3 | 12 Dec 2003 | 126 | Y | |  | |  |  |  |  |  |  |  |  |  |  |  |
| 517-604-4 | 12 Dec 2003 | 126 | Y | |  | |  |  |  |  |  |  |  |  |  |  |  |
| 517-604-5 | 12 Dec 2003 | 126 | n | |  | |  |  |  |  |  |  |  |  |  |  |  |
| 517-604-6 | 12 Dec 2003 | 126 | Y | |  | |  |  |  |  |  |  |  |  |  |  |  |
| 517-605-1 | 12 Dec 2003 | 127 | Y | |  | | 365 | Y |  |  |  |  |  |  |  |  |  |
| 517-605-2 | 12 Dec 2003 | 127 | Y | |  | | 365 | Y |  |  |  |  |  |  |  |  |  |
| 517-605-3 | 12 Dec 2003 | 127 | Y | |  | |  |  |  |  |  |  |  |  |  |  |  |
| 517-605-4 | 12 Dec 2003 | 127 | Y | |  | |  |  |  |  |  |  |  |  |  |  |  |
| 517-605-5 | 12 Dec 2003 | 127 | Y | |  | |  |  |  |  |  |  |  |  |  |  |  |
| 510-602-2a | 21 Jan 2003 | 149 | n | |  | | 314 | Y |  | 367 | Y |  | 2344 | n |  |  |  |
| 510-602-2b | 21 Jan 2003 | 149 | n | |  | |  |  |  |  |  |  |  |  |  |  |  |
| 510-602-2c | 21 Jan 2003 | 149 | n | |  | |  |  |  |  |  |  |  |  |  |  |  |
| 510-602-2d | 21 Jan 2003 | 149 | n | |  | |  |  |  |  |  |  |  |  |  |  |  |
| 510-402-3 | 19 Jan 2003 | 175 | Y | |  | | 948 | Y |  | 1958 | Y |  | 2905 | Y |  |  |  |
| 510-602-3c | 22 Jan 2003 | 203 | n | |  | |  |  |  |  |  |  |  |  |  |  |  |
| 510-602-1a | 21 Jan 2003 | 205 | n | |  | |  |  |  |  |  |  |  |  |  |  |  |
| 510-602-1b | 21 Jan 2003 | 205 | Y | |  | |  |  |  |  |  |  |  |  |  |  |  |
| 510-602-1d | 21 Jan 2003 | 205 | Y | |  | | 315 | n |  | 681 | n |  | 2108 | n |  |  |  |
|  |  |  |  | |  | |  |  |  |  |  |  |  |  |  |  |  |
| Supplemental Table 1c. Transmission attempts for individual *O. tartakovskyi* tested at various days after being orally exposed to Karshi virus | | | | | | | | | | | | | | | | | |
| 510-501-1 | 21 Jan 2003 | 59 | | n | |  |  |  |  |  |  |  |  |  |  |  |  |
| 510-501-2 | 21 Jan 2003 | 59 | | n | |  | 367 | Y |  |  |  |  |  |  |  |  |  |
| 510-701-1 | 22 Jan 2003 | 64 | | n | |  | 2108 | n |  | 2342 | n |  |  |  |  |  |  |
| 510-502-1 | 21 Jan 2003 | 94 | | n | |  |  |  |  |  |  |  |  |  |  |  |  |
| 510-502-2 | 21 Jan 2003 | 94 | | n | |  |  |  |  |  |  |  |  |  |  |  |  |
| 510-502-3 | 21 Jan 2003 | 94 | | n | |  | 2109 | Y |  |  |  |  |  |  |  |  |  |
| 510-701-5 | 22 Jan 2003 |  | |  | |  | 253 | Y |  | 323 | Y |  |  |  |  |  |  |
| 510-701-3 | 22 Jan 2003 |  | |  | |  | 253 | n |  |  |  |  |  |  |  |  |  |
| 510-701-4 | 22 Jan 2003 |  | |  | |  | 366 | Y |  | 1109 | Y |  |  |  |  |  |  |
