# Supplementary material for: Experimental Transmission of Karshi (Mammalian Tick-Borne Flavivirus Group) Virus by Ornithodoros Ticks >2,900 Days after Initial Virus Exposure Supports the Role of Soft Ticks as a Long-Term Maintenance Mechanism for Certain Flaviviruses
Source: PLoS Negl Trop Dis. 2015 Aug 18;9(8):e0004012. doi: 10.1371/journal.pntd.0004012 (PMC4540281; doi:10.1371/journal.pntd.0004012)
Supplement: S2 Table — (DOCX) [file pntd.0004012.s002.docx]

| Supplemental Table 2a. Transmission attempts for individual *O. parkeri* held at various days after being inoculated with Karshi virus | | | | | | | | | |
| --- | --- | --- | --- | --- | --- | --- | --- | --- | --- |
| Tick  number | Date  Inoc. | Day | Trans.  Y/n |  | Day | Trans.  Y/n |  | Day | Trans.  Y/n |
| 517-304-3 | 6 Aug 2003 | 127 | Y |  | 162 | Y |  |  |  |
| 517-304-5 | 6 Aug 2003 | 127 | Y |  |  |  |  |  |  |
| 517-304-6 | 6 Aug 2003 | 127 | Y |  |  |  |  |  |  |
| 517-201-3 | 6 Aug 2003 | 127 | Y |  |  |  |  |  |  |
| 517-202-1 | 6 Aug 2003 | 127 | Y |  | 162 | Y |  |  |  |
| 517-202-2 | 6 Aug 2003 | 1910 | Y |  |  |  |  |  |  |
|  |  |  |  |  |  |  |  |  |  |
| Supplemental Table 2b. Transmission attempts for individual *O. sonrai* held at various days after being inoculated with Karshi virus | | | | | | | | | |
| 510-201-1 | 23 Jan 2003 | 43 | Y |  | 177 | Y |  |  |  |
| 510-201-2 | 23 Jan 2003 | 43 | Y |  |  |  |  |  |  |
| 510-201-3 | 23 Jan 2003 | 43 | Y |  | 203 | Y |  |  |  |
| 510-201-4 | 23 Jan 2003 | 43 | Y |  |  |  |  |  |  |
| 510-201-5 | 23 Jan 2003 | 43 | n |  | 203 | Y |  |  |  |
| 510-201-6 | 23 Jan 2003 | 43 | Y |  |  |  |  |  |  |
| 510-202-3 | 23 Jan 2003 |  |  |  | 64 | Y |  |  |  |
| 510-302-4 | 23 Jan 2003 |  |  |  | 148 | Y |  |  |  |
| 510-302-5 | 23 Jan 2003 |  |  |  | 148 | Y |  |  |  |
| 510-302-6 | 23 Jan 2003 |  |  |  | 148 | Y |  |  |  |
| 510-303-6 | 23 Jan 2003 |  |  |  | 252 | Y |  |  |  |
| 510-602-4 | 23 Jan 2003 |  |  |  | 148 | Y |  | 203 | Y |
| 510-602-5 | 23 Jan 2003 |  |  |  | 148 | Y |  |  |  |
| 510-602-6 | 23 Jan 2003 |  |  |  | 148 | Y |  | 203 | Y |
|  |  |  |  |  |  |  |  |  |  |
| Supplemental Table 2c. Transmission attempts for individual *O. tartakovskyi* held at various days after being inoculated with Karshi virus | | | | | | | | | |
| 510-301-1 | 23 Jan 2003 | 43 | n |  | 203 | Y |  |  |  |
| 510-301-2 | 23 Jan 2003 | 43 | Y |  | 366 | Y |  |  |  |
| 510-301-3 | 23 Jan 2003 | 43 | Y |  | 1268 | Y |  | 2106 | Y |
| 510-301-4 | 23 Jan 2003 | 43 | Y |  |  |  |  |  |  |
| 510-301-5 | 23 Jan 2003 | 43 | Y |  | 366 | Y |  |  |  |
| 510-301-6 | 23 Jan 2003 | 43 | Y |  | 366 | Y |  |  |  |
| 510-302-1 | 23 Jan 2003 | 148 | Y |  |  |  |  |  |  |
| 510-303-1 | 23 Jan 2003 | 252 | Y |  |  |  |  |  |  |
| 510-303-2 | 23 Jan 2003 | 252 | Y |  |  |  |  |  |  |
| 510-303-3 | 23 Jan 2003 | 252 | Y |  | 366 | Y |  | 1268 | Y |
| 510-303-4 | 23 Jan 2003 | 252 | Y |  | 1109 | Y |  | 1268 | Y |
| 510-303-5 | 23 Jan 2003 | 252 | Y |  |  |  |  |  |  |
| 510-502-4 | 23 Jan 2003 | 93 | Y |  | 323 | Y |  |  |  |
| 510-502-5 | 23 Jan 2003 | 93 | Y |  |  |  |  |  |  |
| 510-502-6 | 23 Jan 2003 | 93 | Y |  |  |  |  |  |  |
| 510-202-6 | 23 Jan 2003 | 366 | Y |  |  |  |  |  |  |
